# Monitoring of microbial hydrocarbon remediation in the soil

**DOI:** 10.1007/s13205-011-0014-8

**Published:** 2011-07-06

**Authors:** Chioma Blaise Chikere, Gideon Chijioke Okpokwasili, Blaise Ositadinma Chikere

**Affiliations:** 1 Department of Microbiology, University of Port-Harcourt, P.M.B. 5323, Port Harcourt, Rivers State Nigeria; 2 Shell Petroleum Development Company, P. O. Box 230, Port Harcourt, Rivers State Nigeria

**Keywords:** Bioremediation, Hydrocarbon, Microbial diversity, Molecular techniques

## Abstract

Bioremediation of hydrocarbon pollutants is advantageous owing to the cost-effectiveness of the technology and the ubiquity of hydrocarbon-degrading microorganisms in the soil. Soil microbial diversity is affected by hydrocarbon perturbation, thus selective enrichment of hydrocarbon utilizers occurs. Hydrocarbons interact with the soil matrix and soil microorganisms determining the fate of the contaminants relative to their chemical nature and microbial degradative capabilities, respectively. Provided the polluted soil has requisite values for environmental factors that influence microbial activities and there are no inhibitors of microbial metabolism, there is a good chance that there will be a viable and active population of hydrocarbon-utilizing microorganisms in the soil. Microbial methods for monitoring bioremediation of hydrocarbons include chemical, biochemical and microbiological molecular indices that measure rates of microbial activities to show that in the end the target goal of pollutant reduction to a safe and permissible level has been achieved. Enumeration and characterization of hydrocarbon degraders, use of micro titer plate-based most probable number technique, community level physiological profiling, phospholipid fatty acid analysis, 16S rRNA- and other nucleic acid-based molecular fingerprinting techniques, metagenomics, microarray analysis, respirometry and gas chromatography are some of the methods employed in bio-monitoring of hydrocarbon remediation as presented in this review.

## Introduction

Environmental pollutants can either be organic or inorganic. Quantitatively, the organic pollutants of most concern are hydrocarbons in their various forms. The most common are petroleum hydrocarbons which include *n*-alkanes and other aliphatics, aromatic compounds and other minor constituents (Atlas and Philp 2005; Sarkar et al. 2005). The refining, storage and distribution of crude oil and allied petroleum products are all point sources of soil and water pollution (van Hamme et al. 2003; Chikere et al. 2009a, b; Nogales et al. 2011). Biodegradability of hydrocarbons and hence their degree of persistence in natural environments are influenced by various factors, most vital of which are the chemical structure of the hydrocarbons, the presence of viable microbial population able to degrade them and environmental conditions optimal for microbial degradative activities (Bundy et al. 2002, 2004; Stroud et al. 2007). The process of bioremediation defined as the use of microbes to detoxify or remove pollutants, which relies upon microbial enzymatic activities to transform or degrade offending contaminants, has been greatly used in hydrocarbon mitigation (Roling et al. 2004; Margesin et al. 2007; Wolicka et al. 2009). Bioremediation, especially when it can be carried out in situ, offers a cost-effective means of pollutant cleanup. It is an enhancement of the natural fate of biodegradable pollutants and therefore a green solution to the problem of environmental pollution with little or no ecological impact (Cappello et al. 2007; Kumar and Khanna 2010). The end products of ultimate biodegradation (also known as mineralization) such as water and carbon dioxide are innocuous to man and the environment. During hydrocarbon bioremediation, a number of indices are monitored to score the effectiveness of the technology. Use of fundamental chemical analyses for pollutant identification and standard microbiological techniques for quantification of viable microbial populations are the starting points of monitoring. First, the nature of the contaminants must be determined in terms of concentration and chemical nature followed by the nature of the contaminated matrix. Another parameter that is critical during monitoring is the measurement of microbial populations involved in the degradation and environmental factors that influence the rates of microbial metabolism.

## Interactions between soil and hydrocarbons

Crude oil is a complex mixture of hydrocarbons and oil recovered from different reservoirs, which varies widely in compositional and physical properties. Long recognized as substrates supporting microbial growth (Rosenberg et al. 1996), these hydrocarbons are both targets and products of microbial metabolism (van Hamme et al. 2003). Crude oil hydrocarbons can be divided into four classes: the aliphatics, the aromatics, the asphaltenes and the resins. Hydrocarbons differ in their susceptibility to microbial attack and, in the past, have been generally ranked in the following order of decreasing susceptibility: n-alkanes > branched alkanes > low molecular weight aromatics > cyclic alkanes > high molecular weight aromatics (Leahy and Colwell 1990).

Alkanes can be utilized by a large number of bacteria. The actual chain length is of decisive importance. Lower molecular weight alkanes are easily volatilized while mid-length (C_14_–C_20_) alkanes are non-polar, virtually water-insoluble hydrocarbons with increasing melting and boiling points as carbon number increases within the molecule. Typically, these alkanes have low aqueous solubilities. For instance, hexadecane has a water solubility of 0.9 μg l^−1^ and is a liquid at room temperature. Collectively, these physicochemical properties mean that mid-length aliphatic hydrocarbons are not readily volatilized or leached from soil. It is well established that interactions between hydrophobic organic contaminants may also be important in controlling their fate and behavior in the soil, involving the interactions with the mineral and organic fractions, which may invariably result in reduction in biological/chemical availability and then allow these contaminants to persist in the soil. Soil is composed of organic and inorganic components separated by pores containing water or air. The interaction between hydrocarbons and mineral surfaces (clay, silt and sand) are only significant when organic matter content is <0.1%. Thus, organic matter is very important in the fate and behavior of organic contaminants. Organic matter is divided into two distinct phases: (1) soft carbon (rubbery) which is the expanded, flexible structures having humic and fulvic acids as key components with sorption described as irreversible, and (2) hard carbon (glassy) defined as rigid, condensed structures with humin, kerogen and pyrogenic carbons as commonly identified components. Sorption of hydrocarbons within the glassy region is characterized by irreversible sequestration (Stroud et al. 2007; van Elsas et al. 2007).

**Fig. 1:**
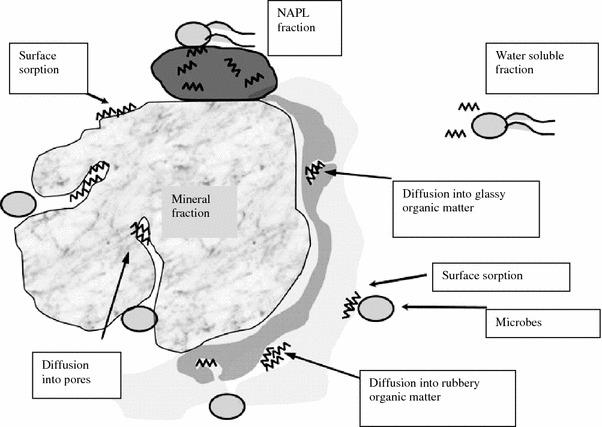
Possible interactions between soil matrices and aliphatic hydrocarbons; NAPL, non aqueous phase liquids (Source: Stroud et al. 2007)

The extent to which a chemical partitions into the organic matter is described by the K_ow_ (octanol–water partition coefficient). Aliphatic hydrocarbons can strongly partition into organic matter and diffuse into the three-dimensional structure of organic matter. Hydrocarbons may be sequestered within the soil through sorption to organic matter and mineral fractions and or diffuse into the three-dimensional structure of the soil (Fig. 1). The degree to which these physical interactions occur increases with time and has been termed ‘aging’. For aromatics, their fate in the soil is dependent to a large extent on their molecular size, i.e., the number of aromatic rings. Generally, an increase in the size and angularity of aromatics results in a concomitant increase in hydrophobicity, electrochemical stability, high sorption capacity and persistence in the soil (Kanaly and Harayama 2000; van Hamme et al. 2003).

## Interaction between microorganisms and hydrocarbons in the soil

The use of hydrocarbons as substrates for microbial growth presents special problems to both the microorganisms using them as a source of carbon and energy and to researchers in the field of petroleum microbiology. There are two essential characteristics that define hydrocarbon-oxidizing microorganisms:

1. membrane-bound, group-specific oxygenases and
2. mechanisms for optimizing contact between the microorganisms and the water-insoluble hydrocarbons.

The localization of hydrocarbon-oxidizing bacteria in natural environments has received considerable attention because of the possibility of utilizing their biodegradation potentials in the treatment of oil spills. Because of the enormous quantities of crude and refined oils that are transported over long distances and consumed in large amounts, hydrocarbons have now become a very important class of substrates for microbial oxidation (Rosenberg and Ron 1996).The biological fate of hydrocarbons in the soil can follow a number of routes. Under ideal conditions, the hydrocarbons are completely mineralized to carbon dioxide and water, with some biomass production. More often, biodegradation is not complete. Microbial metabolism of hydrocarbons to partially oxidized products also serves to remediate polluted soil by fixing and stabilizing potentially hazardous materials. Partially degraded hydrocarbons may become incorporated into the soil organic matter or be humified (Bossert and Compeau 1995). For effective biodegradation to occur, it is therefore essential that the hydrocarbon substrate be ‘bioavailable’ to the degrading microbial communities (Leahy and Colwell 1990; Rosenberg et al. 1996; van Hamme et al. 2003). Understanding the term ‘bioavailability’ is complicated because of a number of interpretations in published literature. In an attempt to clarify this matter, Semple et al. (2004) reported two linked definitions, bioavailability and bioaccessibility. A bioavailable compound is defined as one which is freely available to cross an organism’s membrane from the medium the organism inhabits at a given point in time, while a bioaccessible compound is described as a compound which is available to cross an organism’s membrane from the environment it inhabits, if the organism has access to it. However, it may either be physically removed from the organism, or only bioavailable after a period of time. In considering the methods used to determine microbial degradation as well as the use of chemical extractions to predict microbial degradation in published literature, it is reasonable to presume that the latter definition (bioaccessibility) is more likely to be measured both biologically and chemically.

## Microbial ecology of hydrocarbon degradation in the soil

The ability to metabolize hydrocarbons is displayed by many different types of microbes. There is a myriad of literature on the subject of hydrocarbon degradation by microorganisms and it is now generally accepted that no single species will completely degrade any complex class of hydrocarbons. Although it is widely accepted that bacteria and fungi are primary mediators in hydrocarbon degradation, bacteria have been shown to be more versatile than fungi and therefore may play a greater role during biodegradation of hydrocarbons. Based on published reports, the most important hydrocarbon-degrading bacterial genera in soil environments include *Achromobacter*, *Acinetobacter*, *Alcaligenes*, *Arthrobacter*, *Bacillus*, *Burkholderia*, *Collimonas*, *Corynebacterium*, *Dietzia*, *Flavobacterium*, *Gordonia*, *Micrococcus*, *Mycobacterium*, *Nocardia*, *Nocardioides*, *Pseudomonas*, *Ralstonia*, *Rhodococcus*, *Sphingomonas*, *Variovorax* and other unculturable bacterial clones (Leahy and Colwell 1990; Hamamura et al. 2006; Chikere et al. 2009a; Obayori and Salam 2010). Among the fungi, *Aspergillus*, *Candida*, *Cunninghamella*, *Fusarium*, *MucorPenicillium*, *PhanerochaeteRhodotorula*, *Sporobolomyces* and *Trichoderma* are hydrocarbon-degrading genera frequently isolated from soil. Fungal hyphal structures and increased surface area allow for better penetration and contact with hydrocarbons. Their extracellular enzymes, e.g., oxidases may further extend their activity into the soil (Young and Cerniglia 1995). Prior exposure to hydrocarbons results in adaptation of the microbial community to utilize hydrocarbons as carbon and energy sources. The three interrelated means by which adaptation can occur are (1) induction and or depression of specific enzymes, (2) genetic changes which result in metabolic pathways and (3) selective enrichment of microbes able to transform the hydrocarbons (Leahy and Colwell 1990). Bacteria exhibit these phenomena more than any other microbial group after hydrocarbon perturbation in the soil. Some of the hydrocarbon degradation capabilities that exist in bacteria include possession of degradative plasmids and other mobile genetic elements (Rojo 2009), surfactant production (Van Hamme et al. 2003) and possession of specific catabolic enzymes like oxygenases and hydoxylases (Atlas and Philp 2005). Horizontal gene transfer is more widespread in bacteria and has been reported as one of the major mechanisms responsible for the evolution of enhanced hydrocarbon degradation (Obayori and Salam 2010). Flocco et al. (2009) investigated the diversity of naphthalene dioxygenase genes in soils from Maritime Antarctic using nested PCR, and DGGE cloning and sequencing. Their study revealed the predominance of nahAc-like genes carried on *Pseudomonas*-associated plasmids in the microbial communities from the hydrocarbon-contaminated soil and quantitative PCR also indicated that their relative abundance increased in response to anthropogenic contamination.

## Biodegradation of hydrocarbons in the soil

Crude oils are composed of complex mixtures of paraffinic, alicyclic and aromatic hydrocarbons. A terrestrial oil spill left to its natural fate is gradually degraded by some biological and non-biological mechanisms (Leahy and Colwell 1990; Atlas and Bartha 1998; van Hamme et al. 2003). Photooxidation may contribute substantially to the self-purification of the soil. Laboratory experiments suggest that in 8 h of effective sunshine, as much as 0.2 metric ton of oil per square kilometer may be destroyed by photooxidation (Atlas and Bartha 1998). Communities exposed to hydrocarbon become adapted exhibiting selective enrichment and genetic changes resulting in increased proportions of hydrocarbon-degrading bacteria and bacterial plasmids encoding hydrocarbon catabolic genes (Leahy and Colwell 1990; Rosenberg and Ron 1996; Quatrini et al. 2008).

**Fig. 2:**
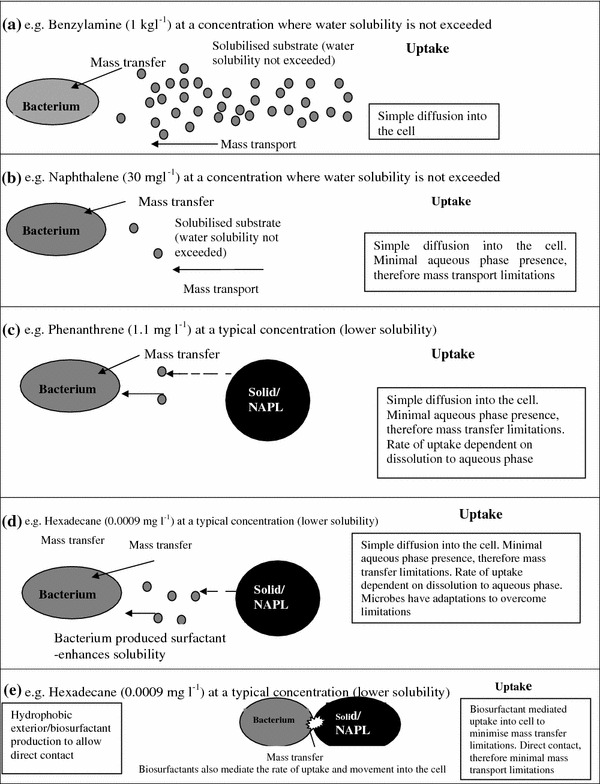
Range of bacterial uptake mechanisms for hydrocarbons in the soil (Source: Stroud et al. 2007)

Because adapted microbial communities have higher proportions of hydrocarbon degraders, they can respond to the presence of hydrocarbon pollutants within hours (Atlas and Bartha 1998; van Elsas et al. 2007). A number of uptake mechanisms might be employed by acclimatized microbes during the biodegradation of different components of crude oil as illustrated in Fig. 2. The majority of petroleum hydrocarbons are very hydrophobic and this limits the capacity of microbes, which generally exist in aqueous phase, to access and degrade them. Hydrocarbon-degrading microbes can overcome this by producing biosurfactants (van Hamme et al. 2003). For medium and long chain length alkanes, microorganisms may gain access either by adhering to hydrocarbon droplets or by surfactant-facilitated process. Most alkane-degrading bacteria secrete diverse surfactants that facilitate emulsification of the hydrocarbons. Surfactants produced by microbes probably have other roles as well, such as facilitating cell motility on solid surfaces or the adhesion/detachment to surfaces or biofilms (Rojo 2009). The Gram-negative bacterium *Acinetobacter* is widely known to produce biosurfactants/bioemulsifiers; thus it has a hydrophobic exterior to allow cellular contact with hydrocarbons during biodegradation (Stroud et al. 2007)

## Aliphatic hydrocarbons

**Fig. 3:**
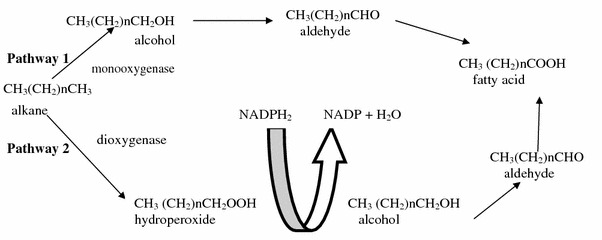
Biodegradation of alkanes (Source: van Elsas et al. 2007)

Complete degradation of aliphatic hydrocarbons results in the formation of carbon dioxide and water. There are two biodegradation pathways for the alkanes. The initial step in the aerobic degradation of saturated, aliphatic hydrocarbons (*n*-alkanes) involves the enzymes that have a strict requirement for molecular oxygen, i.e., monooxygenases (mixed function oxidases) or dioxygenases. The most common pathway depends on the action of monooxygenase enzymes and is specific for *n*-alkanes (Stroud et al. 2007). The monooxygenase attacks the terminal methyl group where a primary alcohol is formed (van Hamme et al. 2003). The alcohol is further oxidized to the corresponding aldehyde and fatty acid (pathway 1; Fig. 3).

In the second pathway (Fig. 3), a dioxygenase enzyme acts on the terminal methyl group of an *n*-alkane resulting in the addition of two oxygen atoms. This results in the formation of a peroxide that is converted to a fatty acid. The carboxylic acid groups in the fatty acids resulting from either pathway are then further metabolized via the β-oxidation pathway (Fig. 3), a common catabolic pathway found in most living cells, to form acetyl CoA or propionyl CoA [depending on the number of carbon atoms (even or odd) in the *n*-alkane]. These compounds are then subsequently metabolized via the tricarboxylic acid cycle (TCA cycle) to CO_2_ and H_2_O (Atlas and Bartha 1998; van Hamme et al. 2003; van Elsas et al. 2007).

## Aromatic hydrocarbons

**Fig. 4:**
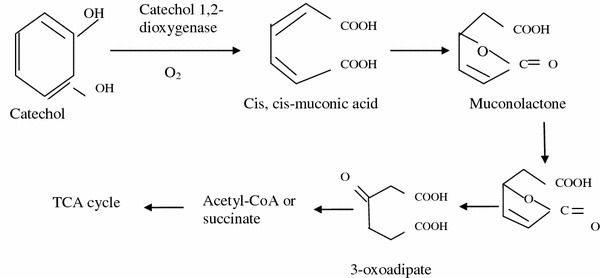
Ortho-cleavage of catechol in the TCA (tricarboxylic acid) cycle (Source: van Elsas et al. 2007)

**Fig. 5:**
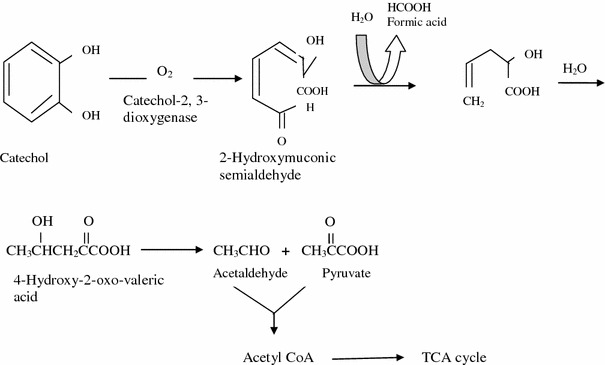
Meta-cleavage of catechol in the TCA (tricarboxylic acid) cycle (Source: van Elsas et al. 2007)

**Fig. 6:**
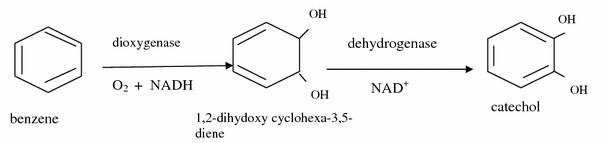
Biotransformation of benzene to catechol. (Source: van Elsas et al. 2007)

Low solubility is one reason for the low rate of biodegradation of aromatic hydrocarbons in addition to problems due to production of toxic dead-end metabolites, metabolite repression, presence of preferred substrates and lack of co-metabolic substrates (Kanaly and Harayama 2000; van Hamme et al. 2003; van Elsas et al. 2007). While some simple aromatic hydrocarbons (e.g., BTEX) are present in petroleum, naphthalene, with two aromatic rings, represents the simplest polycyclic aromatic hydrocarbon (PAH). Among the PAHs, the low molecular weight hydrocarbons (<3 rings) are more susceptible to microbial degradation than the high molecular weight PAHs (>4 rings) (Kanaly and Harayama 2000; van Elsas et al. 2007). To date, no single microorganism has been reported that can utilize high molecular weight PAHs such as benzo(*a*)pyrene as the sole source of energy, although transformations through co-metabolic activities have been reported (van Elsas et al. 2007). Biodegradation mechanisms require the presence of molecular oxygen to initiate the enzymatic attack of PAH rings. In the first step, dioxygenase-catalyzed oxidation of arenes generally takes place in aerobic bacteria to yield vicinal *cis*-dihydrodiols as early intermediates by a multicomponent enzyme system. These dihdroxylated by-products may then be cleaved by intradiol or extradiol ring-cleaving dioxygenases through either an ortho-cleavage pathway (Fig. 4) or meta-cleavage pathway (Fig. 5), leading to intermediates such as protocatechuate and catechols (Fig. 6; using benzene as an example) (Peng et al. 2008)

Using benzene catabolism as an example (Fig. 6), a dioxygenase facilitates the oxidation of benzene to benzene dihydrodiol, which is subsequently converted to catechol. The catechol aromatic ring is then cleaved, again with the help of a dioxygenase. Catechol catabolism can subsequently follow one of two pathways as described above:

1. The *ortho*-cleavage pathway, in which the ring is cleaved between the two carbon atoms with hydroxyl groups (Fig. 4).
2. The *meta*-cleavage pathway, in which the ring splits between adjacent carbon atoms with and without a hydroxyl group (Fig. 5).

Breakdown metabolites such as acetate, succinate, pyruvate or acetaldehyde from either of the pathways subsequently enter the TCA cycle and are thus available as energy and carbon sources to the cell (Atlas and Bartha 1998; Kanaly and Harayama 2000; Schlegel 2002). Microbial degradation of crude oil has been shown to occur by attack on the aliphatic or aromatic fractions. Although some studies have reported their removal at high rates under optimal conditions (Leahy and Colwell 1990; Rosenberg et al. 1992; Atlas and Bartha 1998; Margesin et al. 2003; Hamamura et al. 2006; Maila et al. 2006, 2008), high molecular weight aromatics, resins and asphaltenes are generally considered to be recalcitrant or exhibit only slow rates of biodegradation (Atlas and Bartha 1998; van Hamme et al. 2003; Stroud et al. 2007).

## Soil microbial (bio) diversity

Biological diversity or biodiversity is defined as the collective variation at all levels of biological organization, from the genetic variations within populations and species, species within communities, to communities that compose an ecosystem (Torsvik and Øvreås 2007). While microbial diversity on the other hand can be defined as the variety of bacterial species in ecosystems as well as the genetic variability within each species. Biodiversity commonly describes both the information content and how this information is distributed, in biological systems such as microbial assemblages. Taking into account the small sizes of microorganisms, one soil particle of just 1–2 mm dimension can be compared to a whole landscape for higher organisms and, as such, the origin of the tremendous microbial diversity in the soil and its relation to the processes mediated by the indigenous microbial communities is still poorly understood (Torsvik and Øvreås 2007). Because of this immense genotypic and phenotypic diversity, microbial communities in the soil remain some of the most difficult to characterize (Atlas and Bartha 1998; Maila 2005; Smalla et al. 2007; Malik et al. 2008). Two approaches generally used to characterize microbial diversity include culture-dependent (or phenotypic) or culture-independent (genomic) based techniques. However, due to the heterogeneity of the soil and the inherent limitations of the existing traditional methodologies, most of the bacterial species in the soil remain unidentified. According to literature, in soil ecosystem, in which most of the niches or tasks are fulfilled, microbial diversity is high (Ollivier and Magot 2005; Maila 2005; Smalla et al. 2007; Torsvik and Øvreås 2007; Ge et al. 2008). However, the microbial community structure changes and diversity decreases due to environmental stresses or disturbances (Macnaughton et al. 1999; Atlas and Philp 2005). The reports about the relationship between environmental pollution and the shifts in soil microbial communities are well established together with the phenomenon that changes in community structure usually result in the emergence of dominant populations within the disturbed communities that can survive toxic contamination and have enhanced physiological and substrate utilization capabilities (Miller et al. 2002; Margesin et al. 2003; Zucchi et al. 2003; Evans et al. 2004; Alquati et al. 2005; Vinas et al. 2005; Hamamura et al. 2006; Maila et al. 2006; Popp et al. 2006; Smalla et al. 2007; Ge et al. 2008; Rodrigues et al. 2009; Wolicka et al. 2009; Kumar and Khanna 2010).

Microbe-driven functions are responsible for a wide range of nutrient cycling and geochemical processes in soil. Thus, microbial diversity in soil is crucial for soil functioning and health, and so there is a need to understand at community and species levels the spatial and temporal variability of microbial community structure and functions, e.g., in response to crude oil/hydrocarbon pollution (Macnaughton et al. 1999; Smalla et al. 2007). Crude oil pollution can cause microbial community shifts and a decrease in microbial diversity (Rosenberg and Ron 1996; Hamamura et al. 2006). However, as most of the low molecular weight hydrocarbons are volatile and the high molecular weight alkanes are biodegradable, the impact of hydrocarbons on microbial diversity in soil can be mitigated by bioremediation (Leahy and Colwell 1990; Kaplan and Kitts 2004; Philp et al. 2005). It is therefore important to understand the microbial community structure in the soil, as existing bioremediation technologies can be optimized if the current knowledge of both functional and genetic variability can be improved (Maila 2005; Maila et al. 2006; Lal et al. 2010).

## Methods for monitoring microbial diversity in soil during bioremediation

For bioremediation to be considered a remediation technology, it is critical to establish that there is an adequate active microbial population that is capable of attacking the specific contaminants (Macnaughton et al. 1999; Chaillan et al. 2004; Maila et al. 2006; Wolicka et al. 2009). If the site has the requisite values for water content and pH, has porosity within the desirable range, and is contaminated with only petroleum hydrocarbons, then there is a very good chance that there will be an active population of hydrocarbon-utilizing bacteria in the soil and that bioremediation may be able to succeed (Atlas and Philp 2005; Ollivier and Magot 2005). Use of fundamental chemical analyses for pollutant identification and standard microbiological techniques for quantification of viable populations of microorganisms are the starting points for monitoring. The techniques for determining the presence of hydrocarbon-utilizing microorganisms are routine, inexpensive and relatively rapid. Even when it is difficult to cultivate specific microbial populations, however, the technique of enrichment culture can reveal the presence of important degrading microbes and establish that they have the natural propensity to degrade the pollutant at an acceptable rate of performance (Williams et al. 1999; Atlas and Philp 2005). There are also emerging techniques of molecular microbial ecology that do not rely on cultivation because of the viable, but nonculturable, phenomenon, which has been found to be very useful for monitoring the progress of bioremediation (Siciliano et al. 2003; Kloos et al. 2006; Brons and van Elsas 2008; Malik et al. 2008; Zengler 2008). During bioremediation, microbial population changes can be investigated, along with more detailed analytical work, e.g., gas chromatograph [(equipped with either of the detectors: flame ionization detector (FID) or electron capture detector (ECD)], high performance liquid chromatography, tests on the fate of ^14^C-radiolabeled substrates to identify specifically whether biodegradation and/or mineralization of the substrate is taking place or a mere transformation to a more or less toxic, more or less mobile metabolite (Okpokwasili et al. 1986; Young and Cerniglia 1995; Atlas and Philp 2005). A number of laboratory- and field-scale bioremediation trials have employed these strategies and found them useful in monitoring the progress of bioremediation in different environmental media (Rosenberg et al. 1992, 1996; Rosenberg and Ron 1996; Macnaughton et al. 1999; Odokuma and Ibor 2002; Odokuma and Dickson 2003; Zucchi et al. 2003; Chaillan et al. 2004; Evans et al. 2004; Ebuehi et al. 2005; Maila et al. 2006; Ayotamuno et al. 2006; Adoki and Orugbani 2007; Stroud et al. 2007; Chikere et al. 2008a, b, Chikere et al. 2009c; Popp et al. 2006; Wolicka et al. 2009).

## Microbiological methods

### Monitoring hydrocarbon-utilizing bacteria (HUB)

Crude oil and other petroleum hydrocarbons are chemically heterogeneous and almost ubiquitous in the environment. Not only are they found at the site of oil pollution, but chemical analysis has revealed the presence of both aliphatic and aromatic hydrocarbons in most pristine soils and sediments (Heiss-Blanquet et al. 2005; Ollivier and Magot 2005; Philp et al. 2005; Quatrini et al. 2008). The probable origins of these low concentrations of hydrocarbons in pristine environmental media are seepage from natural deposits and biosynthesis by plants and microorganisms (Atlas and Philp 2005; Ollivier and Magot 2005). It is therefore not surprising that HUB are widely distributed in nature. Several investigations have demonstrated an increase in the number of HUB in oil-polluted habitats undergoing bioremediation (Rosenberg et al. 1992, 1996; Rosenberg and Ron 1996; Bouchez-Naitali et al. 1999; Macnaughton et al. 1999; Williams et al. 1999; Odokuma and Ibor 2002; Margesin et al. 2003; Odokuma and Dickson 2003; Koren et al. 2003; Sei et al. 2003; Siciliano et al. 2003; Bordenave et al. 2004a, b; Chikere and Chijioke-Osuji. 2006; Hamamura et al. 2006; Rojas-Avelizapa et al. 2007; Quatrini et al. 2008; Ruberto et al. 2009). However, previous and recent studies have suggested that despite an increase in the HUB percentage, the biodiversity of the bacterial community may be dramatically reduced, since the presence of hydrocarbons in the environment often leads to selective enrichment of HUB, to the relative detriment of biodiversity (Leahy and Colwell 1990; Rosenberg and Ron 1996; Abed et al. 2002; Evans et al. 2004; Atlas and Philp 2005; Maila et al. 2005; Hamamura et al. 2006; Popp et al. 2006; Quatrini et al. 2008; Rodrigues et al. 2009). To achieve hydrocarbon utilization by bacteria, a number of rate limiting nutritional requirements need to be provided. Hydrocarbons, as their name implies, are composed of hydrogen and carbon; therefore, there is a need to supply all other elements essential for growth in the growth medium (Philp et al. 2005). These growth factors include molecular oxygen for the oxygenases, nitrogen, phosphorus, sulfur and metals like K^+^ and Na^+^ (Leahy and Colwell 1990; Venosa et al. (1996): Atlas and Bartha 1998; Rosenberg et al. 1998; van Hamme et al. 2003; Ollivier and Magot 2005).

The ability to degrade and/or utilize hydrocarbons is exhibited by a wide variety of bacterial genera (Leahy and Colwell 1990). In all, according to Prince (2005), more than 75 validly described genera of bacteria degrade oil and other hydrocarbons in different environmental media. Degraders have been isolated from soils (Bouchez-Naitali et al. 1999; Macnaughton et al. 1999; Williams et al. 1999; Odokuma and Dickson 2003; Siciliano et al. 2003; Zucchi et al. 2003; Chaillan et al. 2004; Evans et al. 2004; Nweke and Okpokwasili 2004; Kaplan and Kitts 2004; Alquati et al. 2005; Ebuehi et al. 2005; Vinas et al. 2005; Ayotamuno et al. 2006; Hamamura et al. 2006; Maila et al. 2006; Adoki and Orugbani 2007; Rojas-Avelizapa et al. 2007; Wolicka et al. 2009), oceans, coastal waters, freshwater and marine sediments, lakes, ponds and estuaries (Leahy and Colwell 1990; Rosenberg et al. 1996; Swannell et al. 1996; Watanabe et al. 2000, 2001; Abed et al. 2002; Abu and Chikere 2006; Kasai et al. 2002; Roling et al. 2002; Koren et al. 2003; Sei et al. 2003; Bordenave et al. 2004a, b; Roling et al. 2004; Prince 2005; Prince and Atlas 2005; Allen et al. 2007; Gontang et al. 2007; Yakimov et al. 2007; Quatrini et al. 2008; Qiao and Shao 2010). Hydrocarbon degraders have also been isolated from temperate (Hamamura et al. 2006; Philp et al. 2005), tropical (Okpokwasili and Odokuma 1994; Odokuma and Ibor 2002; Ijah and Antai 2003a, b; Odokuma and Dickson 2003; Chaillan et al. 2004; Nweke and Okpokwasili 2004; Chikere and Chijioke-Osuji 2006; Okpokwasili 2006) and Arctic environments (Margesin et al. 2003; Ruberto et al. 2009). Hydrocarbon-degrading bacteria known to grow on various hydrocarbons including crude oil are presented in Table 1.

**Table 1:** Genera of bacteria that utilize hydrocarbons as sole source of carbon and energy

Genus	Typical substrate	Degradative genes/enzymes
*Achromobacter*	Gas oil	Alkane hydroxylases
*Acidecella*	Naphthalene	Plasmid-borne genes for dioxygenase
*Acidovorax*	Phenanthrene	Plasmid-borne genes for dioxygenase
*Acinetobacter*	Gas oil	Alkane hydroxylases
*Actinomyces*	Crude oil	Alkane hydroxylases
*Aeromonas*	Diesel oil	Alkane hydroxylases
*Agrobacterium*	Gasoline aromatics	Plasmid-borne genes for dioxygenase
*Alcaligenes*	Gas oil	Plasmid-borne genes for dioxygenase
*Alcanivorax*	Alkanes; crude oil	Alkane hydroxylases
*Alkanindiges*	Alkanes	Alkane hydroxylases
*Alteromonas*	Crude oil	Alkane hydroxylases
*Arthrobacter*	Gas oil	Alkane hydroxylases
*Aureobacterium*	Crude oil	Alkane hydroxylases
*Azoarcus*	Toluene	Plasmid-borne genes for dioxygenases
*Azospirillum*	Jet fuel	Alkane hydroxylases
*Azotobacter*	Crude oil	Alkane hydroxylases
*Bacillus*	Toluene; crude oil	Alkane hydroxylases; plasmid-borne genes for dioxygenases
*Beijerinckia*	Phenanthrene	Plasmid-borne genes for dioxygenases
*Blastochloris*	Toluene	Plasmid-borne genes for dioxygenases
*Brevibacterium*	Alkanes	Alkane hydroxylases
*Brevundimonas*	Fuel oil	Alkane hydroxylases
*Burkholderia*	Toluene	Alkane hydroxylases; plasmid-borne genes for dioxygenases
*Clavibacter*	Naphthalene	
*Comamonas*	Phenanthrene	Plasmid-borne genes for dioxygenase
*Corynebacterium*	Fuel oil; crude oil	Alkane hydroxylases
*Cyclostaticus*	Biphenyl; crude oil	Plasmid-borne genes for dioxygenases
*Cytophaga*	Crude oil	Alkane hydroxylases
*Dechloromonas*	Benzene	
*Desulfatibacillum*	Alkanes	Enzymology not well understood; alkane activation by addition of fumarate terminally/subterminally
*Desulfobacterium*	Xylene	Chromosomally borne benzyl succinate synthase
*Desulfobacula*	Toluene	Chromosomally borne benzyl succinate synthase
*Desulfosarcina*	Xylene	Chromosomally borne benzyl succinate synthase
*Desulfosporosinus*	Gasoline	Enzymology not well understood; alkane activation by addition of fumarate terminally/subterminally
*Dietzia*	Alkanes	Alkane hydroxylases
*Enterobacter*	Alkanes	Alkane hydroxylases
*Erwinia*	Alkanes	Alkane hydroxylases
*Flavobacterium*	Diesel oil; crude oil; phenanthrene	Alkane hydroxylases; plasmid-borne genes
*Geobacillus*	Crude oil	Alkane hydroxylases
*Geobacter*	Toluene	Plasmid-borne genes for dioxygenases
*Gordonia*	Alkanes; crude oil	Alkane hydroxylases
*Klebsiella*	Crude oil	Plasmid-borne genes for dioxygenases
*Lactobacillus*	Crude oil	Alkane hydroxylases
*Leclerica*	Pyrene	Plasmid-borne genes for dioxygenases
*Leucothrix*	Crude oil	Alkane hydroxylases
*Lutibacterium*	Phenanthrene	Plasmid-borne genes for dioxygenases
*Marinobacter*	Crude oil	Alkane hydroxylases
*Micrococcus*	Hexadecane	Alkane hydroxylases
*Moraxella*	Biphenyl	Plasmid-borne genes for dioxygenases
*Mycobacterium*	Phenanthrene	Plasmid-borne genes for dioxygenases
*Neptumonas*	Naphthalenes	Plasmid-borne genes for dioxygenases e.g., NAH7 plasmid and its genes
*Nocardia*	Alkanes; crude oil	Alkane hydroxylases
*Nocardioides*	Phenanthrene; crude oil	Alkane hydroxylases
*Ochrabactrum*	Diesel	Alkane hydroxylases
*Oleiphilus*	Alkanes	Alkane hydroxylases
*Oleispira*	Alkanes; crude oil	Alkane hydroxylases
*Paenibacillus*	Phenanthrene	Plasmid-borne genes for dioxygenases
*Pasteurella*	Fluoranthene	Plasmid-borne genes for dioxygenases
*Peptococcus*	Crude oil	Alkane hydroxylases
*Planococcus*	Alkanes; crude oil	Alkane hydroxylases
*Polaromonas*	Naphthalene	Plasmid-borne genes for dioxygenases e.g., NAH7 plasmid and its genes
*Proteus*	Crude oil	Alkane hydroxylases
*Pseudomonas*	Gas oil; crude oil	Alkane hydroxylases; plasmid-borne genes for dioxygenase
*Ralstonia*	Toluene	Plasmid-borne genes for dioxygenase
*Rhodococcus*	Phenanthrene; crude oil	Alkane hydroxylases
*Sarcina*	Crude oil	Alkane hydroxylases
*Serratia*	Crude oil	Alkane hydroxylases
*Sphaerotilus*	Crude oil	Alkane hydroxylases
*Sphingomonas*	Toluene	Plasmid-borne genes for dioxygenases
*Spirillum*	Crude oil	Alkane hydroxylases
*Staphylococcus*	Diesel	Alkane hydroxylases
*Stenotrophomonas*	Pyrene	Plasmid-borne genes for dioxygenases
*Streptomyces*	Alkanes	Alkane hydroxylases
*Thalossolituus*	Alkanes; crude oil	Alkane hydroxylases
*Thauera*	Toluene	Plasmid-borne genes for dioxygenases
*Thermoleophilum*	Alkanes	Alkane hydroxylases
*Thermoleophilum*	Alkanes	Alkane hydroxylases
*Thermus*	Pyrene	Plasmid-borne genes for dioxygenases
*Terrabacter*	Fluorene	Plasmid-borne genes for dioxygenases
*Vibrio*	Phenanthrene	Plasmid-borne genes for dioxygenases
*Xanthobacter*	Dibenzothiophene	Plasmid-borne genes for dioxygenases closely related to NAH7
*Xanthomonas*	Phenanthrene	Plasmid-borne genes for dioxygenases

Sources: Okpokwasili et al. 1986; Watanabe and Hamamura 2003; Ollivier and Magot 2005; Kloos et al. 2006; Peng et al. 2008; Flocco et al. 2009; Obayori and Salam 2010)

The technique chosen for isolation of HUB depends on the hydrocarbons in question. Isolation techniques have in common their need for a solid surface upon which discrete colonies of bacteria can grow. This is important to obtain axenic cultures. Therefore, the starting point is the need for an agar-based mineral salt medium for isolation (Bouchez-Naitali et al. 1999; Daly et al. 1997; Margesin et al. 2003; Amouric et al. 2006; Hamamura et al. 2006; Rodrigues et al. 2009; Ruberto et al. 2009; Wolicka et al. 2009). To specifically isolate HUB, the hydrocarbons must be provided in the growth medium as the sole source of carbon and energy and no other preferable source (Leahy and Colwell 1990; Odokuma and Dickson 2003; Chikere and Chijioke-Osuji. 2006; Atlas and Philp 2005). A very useful mineral salt medium for HUB enumeration and isolation is Bushnell Haas (BH) agar and it has been used by a couple of researchers for this purpose (Evans et al. 2004; Quatrini et al. 2008).

### Enumeration and isolation of culturable hydrocarbon-utilizing bacteria (HUB)

It is generally assumed that hydrocarbon-contaminated soil can have high concentrations of hydrocarbons which represent high-calorie sustenance for bacteria; then during bioremediation, there should be a mass proliferation of bacteria. This assumption could be wrong. Before a heterotrophic bacterium can start to grow and multiply, it must expend energy for maintenance of viability, called maintenance energy. This energy is derived from the oxidation of organic compounds. When the supply of the organic compound is large, then total available energy will be in excess of maintenance energy, and therefore growth and multiplication can take place (Atlas and Philp 2005). However, since most hydrocarbons are hydrophobic, the actual supply of the carbon is limited by diffusion from the liquid or solid state into the cell surface (Rosenberg and Ron 1996; Rosenberg et al. 1998; Atlas and Philp 2005). When bioavailable carbon is low in concentration, a relatively larger amount of that carbon is required for maintenance energy and as such there will be a smaller amount available for cell growth and division (van Hamme et al. 2003; Atlas and Philp 2005). This phenomenon is also a reason why bioaugmentation with large number of bacterial cells is unsuccessful; if the introduced bacteria are limited in bioavailable carbon and they have a likelihood of starving to death (Leahy and Colwell 1990; Macnaughton et al. 1999; Odokuma and Dickson 2003; Philp et al. 2005). According to Alexander (1999), at threshold level, all the carbon that reaches and enters the cell will be used for maintenance and none is available for growth and division, thus analytical chemistry results at this juncture will demonstrate contaminant attenuation, whereas microbiological results will show that the population and biomass are not increasing.

A common technique for the isolation of crude oil-utilizing bacteria from oil-contaminated soil is the vapor phase transfer in which a mineral salts agar is inoculated with known aliquot of the soil suspension and a filter paper soaked with the crude oil or other hydrocarbons is placed in the lid of the Petri dish (Hamamura et al. 2006; Quatrini et al. 2008). According to Philp et al. (2005), an obvious limitation of this technique is that only a proportion of the total bacterial population that can utilize the vapor phase hydrocarbons can be isolated. This method is likely to underestimate the total crude oil-degrading populations. However, a further criticism of this method is that it cannot be assumed that colonies that appear on the agar plates are really oil degraders, as agar can contain some impurities to allow microbial growth; agar can also absorb volatile nutrients from the air in amounts sufficient to support the growth of many non-oil degrading bacteria (van Hamme et al. 2003; Philp et al. 2005). Hamamura et al., (2006) obtained numerous alkane-degrading bacteria using the vapor phase transfer, which did not correspond to prominent DGGE bands in the oil-contaminated soil they studied. They suggested that results from culture-dependent isolation of hydrocarbon-utilizing bacteria should be confirmed using culture-independent molecular techniques such as denaturing gradient gel electrophoresis of polymerase chain reaction-(PCR) amplified genes such as 16S rRNA.

### Enumeration of unculturable HUB

It is estimated that <1% of environmental isolates are culturable. Fast growing organisms or strains best adapted to particular culture conditions grow preferentially more than those which are not and therefore do not accurately represent the actual community composition actively involved in hydrocarbon degradation. Hence, cultivation-dependent characterization of microorganisms during contaminant degradation may limit the scope of microbial biodiversity and the ecological importance of unculturable organisms may go undetected or underestimated (Malik et al. 2008; Rajendhran and Gunasekaran 2011). The genomes of these mainly uncultured species encode a largely untapped reservoir of novel enzymes and metabolic capabilities. Metagenomics bypasses the need for microbial cultivation. Nucleic acid-based methods such as PCR, denaturing gradient/temperature gradient gel electrophoresis (DGGE/TGGE) and other microbial typing methods based on rRNA sequences have been used for functional genomic characterization of hydrocarbon degraders. A summary of the different molecular methods used in microbial monitoring of bioremediation is shown in Table 2. Kloos et al. (2006) employed a PCR hybridization method for the detection of alkane monooxygenase homologous genes (alkB) to analyze the diversity of alkB sequences from different soil samples. They found out that alkB sequences detected in the investigated soil samples showed the highest similarities to alkB from *Nocardioides*, *Mycobacterium* and *Alcanivorax*. Chikere (2010) used PCR-amplification of 16S rRNA followed by DGGE and sequencing to characterize bacteria involved in crude oil biodegradation after nutrient enhancement. The findings of the research revealed that members of the *Actinobacteria* phylogenetic group and some uncultured bacteria were the dominant organisms involved in crude oil biodegradation. A couple of reviews (Watanabe and Hamamura 2003; Peng et al. 2008; Rojo 2009; Obayori and Salam 2010; Nogales et al. 2011) have well articulated cultivation-independent techniques employed in microbial identification during bioremediation of hydrocarbons.

**Table 2:** Molecular methods for monitoring microbial population dynamics and composition during hydrocarbon bioremediation

Method	Principle	Limitations	References
PCR: simple, multiplex and real time	Specific amplification and quantification of target genes of interest	DNA contamination; non-specific primer annealing and presence of PCR inhibitors like humic acids	Zengler 2008
16S rRNA sequencing	Sequencing of PCR-amplified or cloned 16S	Does not have enough discriminatory power for species delineation as complete 16S gene sequence. Presence of mosaicism in 16S genes may lead to misidentification	Snyder and Champness 2007
16S pyrotags	Pyrosequencing of 16S rRNA. High-throughput technique for metagenomic analysis	Gives only a small tag of the 16S gene, about 200 bp	
Amplified ribosomal DNA restriction analysis (ARDRA)	Digestion of PCR-amplified 16S rRNA fragments with restriction enzymes to give microbial community profile	Microbial community fingerprint is highly influenced by the choice of restriction enzymes	Malik et al. 2008
Terminal restriction fragment length polymorphism (T-RFLP)	Modification of ARDRA; but PCR primers used in T-RFLP are fluorescently labeled so PCR products can be visualized and quantified. Polymorphism in the length of fluorescently labeled terminal restriction fragment of 16S gene aids quantification of microbial community	Multiple restriction enzymes are needed to describe microbial population in a sample	Smalla et al. 2007
16S-23S internally transcribed spacer (ITS) typing	Polymorphism in the length, sequences or RFLP pattern in the ITS region. Exhibits greater length and sequence variation than 16S gene sequence. Useful for identification of closely related organisms	Has relatively small database for comprehensive identification of unculturable organisms	Rajendhran and Gunasekaran 2011; Simon and Daniel 2011
Automated ribosomal intergenic spacer analysis (ARISA)	Polymorphism in the length of fluorescently labeled ITS regions. Useful for metagenomic studies	Database not as extensive as 16S database for complete identification of organisms	Rajendhran and Gunasekaran 2011
Denaturing gradient/temperature gradient gel electrophoresis (DGGE/TGGE)	Polymorphism based on the separation of partially melted 16S rRNA in a linear denaturing gradient/temperature gradient. Bands can be excised from gel and sequenced for identification and phylogenetic analysis	Sequence information from microbial population is limited to 500 bp of 16S rRNA	Muyzer and Smalla 1998
Fluorescent in situ hybridization (FISH)	Hybridization with strain/species/group/domain-specific DNA probes in total genomic DNA after treatment of microbial cells with fixatives. Hybridization with rRNA-targeted probes enhances characterization of uncultured microorganisms	Limited number of probes can be used in a single hybridization experiment; background fluorescence in some samples interferes with epifluorescence or confocal laser microscopy hence affects microbial detection; prior knowledge of sample and microorganisms important for design of specific probes; probe permeability	Christensen et al. 1999; van Elsas et al. 2007
Stable isotope probing (SIP)	Incorporation of stable isotope-labeled substrates into cellular biomarkers such as PLFA, rRNA and DNA that can be used to identify organisms assimilating the substrates. It directly links phylogeny with ecological function	May lack sensitivity; enrichment bias may not reflect substrate metabolism in the environment	Neufeld et al. 2007
Reverse sample genome probing (RSGP)	Cross hybridization between isolated microbial chromosomal DNA from pure cultures and standard microbial species. Genomes exhibiting more than 70% cross hybridization are often regarded as the same species. Can be used to identify and characterize bacteria	Not suitable for environment where prior pure culture isolation has not occurred; not applicable to uncultured organisms in an environment	Malik et al. 2008
DAN microarrays	Miniaturized array of complementary DNA probes (500–5,000 nucleotides long) or oligonucleotides (15–17 bp) attached directly to a matrix for simultaneous hybridization of a large set of probes complementary to their corresponding DNA/RNA targets in a sample. Used to identify organisms and define their ecological role	Lack of specificity, sensitivity and quantification	Gentry et al. 2006
Single strand conformation polymorphism (SSCP)	Polymorphism based on the single-stranded 16S rRNA in polyacrylamide gel	Covers only less than 500 bp of 16S gene	Smalla et al. 2007
Genome fragment enrichment (GFE)	DNA fragments are isolated from metagenomic DNA target sample by hybridizing it to another DNA reference sample to which it is being compared. It reduces complexity of the sample and enriches for sequences that are specific to one sample, thus focusing analyses on the difference in the genetic complement of the two environments	Only covers relatively small fragments	Morales and Holben 2011

### Microtiter plate-based MPN techniques

As a result of the limitations of the traditional solid agar-based isolation methods, liquid culture methods were developed by using the most-probable number (MPN) procedure (Mills et al. 1978). MPN is a statistical method based upon dilution of a sample to extinction, i.e., multiple replicates of a sample are analyzed and the results compared with statistical tables to determine the MPN of microorganisms in the original sample. The development of 96-well microtiter plates gave the opportunity to miniaturize the method. The sheen screen method introduced by Brown and Braddock (1990), represented the beginning of the miniaturized MPN method for oil degraders. This method was specific for crude oil as a substrate. Wrenn and Venosa (1996) developed a 96-well microtiter plate MPN procedure to separately enumerate aliphatic and aromatic hydrocarbon degraders in separate plates. The alkane degrader MPN method uses n-hexadecane as the carbon source, while growth is scored by turbidity and the reduction of iodonitrotetrazolium violet to iodonitrotetrazolium fromazan (red precipitate) is used as an indicator of electron transport activity. An inherent limitation in the 96-well microtiter plate MPN method is lack of bioavailability of the hydrocarbon. Because of the small confined nature of the wells, both oxygen mass transfer and mixing of the oil and the aqueous phase are limited. This means that the incubation periods are long (usually 2 weeks) and there may be a high risk of wells drying out or the medium may become very saline through evaporation.

### Use of enzyme activities to isolate hydrocarbon-utilizing bacteria

The enzymes of greatest interest in hydrocarbon degradation are the dioxygenases because of their important roles in substrate activation and aromatic ring cleavage (Atlas and Bartha 1998). Most crude oils contain high concentrations of aromatic hydrocarbons (Ollivier and Magot 2005). Dioxygenase activity can be screened for by the inclusion of indole in mineral salt medium agar plates. Dioxygenases convert indole to indigo and the presence of blue colonies is the selection criterion (Philp et al. 2005). A more specific enzyme screen is for catechol 2,3-dioxygenase. Catechol is an extremely important and common intermediate in aromatic hydrocarbon catabolism (van Elsas et al. 2007). Colonies can be sampled by filter lift from plates sprayed with catechol. The appearance of yellow pigment within 10 min of incubation at room temperature implies catechol 2,3-dioxygenase activity. Alquati et al. (2005) used the catechol color assay to isolate naphthalene degrading bacteria belonging to the genera *Rhodococcus*, *Arthrobacter*, *Nocardia* and *Pseudomonas* from a petroleum-contaminated soil. In more recent times, primers specific for hydrocarbon-degrading enzymes are used in PCR and other fingerprinting methods in order to elucidate the degradative genes in putative hydrocarbon degraders from petroleum-contaminated soil (Zucchi et al. 2003; Alquati et al. 2005; Higashioka et al. 2009).

## Physicochemical methods

### Respirometry

Metabolic gas respirometry is a technique that gives a rate of reaction by measuring either O_2_ consumption or CO_2_ evolution. Radiorespirometry using ^14^C-labeled hydrocarbons, labeled at the most recalcitrant part of the molecule, is more sensitive, yet very expensive, more demanding technically, uses specific hydrocarbons and generates hazardous radioactive waste (Okpokwasili et al. 1986; Philp et al. 2005). Metabolic gas respirometry is much more flexible because of fewer technical constraints associated with this method. However, doubts over the sensitivity of respirometry have been raised especially at sites where the concentration of oxygen is very low. Whereas respirometry is a proven technique for determining biokinetic parameters for biodegradation of contaminants in groundwater, it remains to be successfully used in soils. Oxygen consumption is less sensitive than CO_2_ evolution (Philp et al. 2005) as oxygen consumption may arise from biotransformation and not necessarily mineralization. CO_2_ production, on the other hand, actually provides data on mineralization and is very useful for assessing biodegradability in solid media like soil and sediments (Itavaara and Vikman 1996). Valuable data can be obtained when both O_2_ consumption and CO_2_ evolution are measured simultaneously using a GC equipped with concentric columns (Philp et al. 2005). This procedure permits online monitoring and data obtained can be used to establish O_2_ uptake rate or the CO_2_ evolution rate together with the respiratory quotient (the ratio of CO_2_ produced to O_2_ consumed) (Bellon-Maurel et al. 2003). Okpokwasili et al. (1986) used hexane-dissolved phenanthrene labeled with ^14^C in the C-9 position to investigate the role of plasmids in the degradation of hydrocarbons in estuarine bacteria namely *Escherichia coli* HB101, *Flavobacterium* sp. SB23 and a cured strain of SB23. They found out that plasmid bearing *Flavobacterium* sp. SB23, which produced clear zones on phenanthrene agar, had greater ^14^CO_2_ evolution (which evidenced mineralization) than other bacteria with or without plasmid. Bogan et al. (2003) used measurement of ^14^CO_2_ evolution to monitor the degradation of ^14^C-fluorene and ^14^C-benzo[*a*]pyrene by a strain of *Mycobacterium austroafricanum*. Their findings showed that the production of ^14^C from the radiolabeled hydrocarbons was as a result of mineralization by the bacterium and not due to abiotic factors like photolysis, as the control experiment showed no ^14^C production. Margesin et al. (2003) used the Isermeyer technique to measure soil respiration (CO_2_ evolution) in petroleum hydrocarbon-contaminated Alpine soils. They used this method to quantify CO_2_ produced during a 24-h period by titration. Their results correlated positively with the microbiological data obtained attributing the hydrocarbon attenuation observed to microbial activities rather than abiotic losses. In the same vein, Siciliano et al. (2003) used ^14^C-labeled hexadecane, naphthalene and phenanthrene to assess the potential of soil microorganisms to degrade these hydrocarbons during a phytoremediation field trial. They recorded significant mineralization around the rhizospheres of selected plants used in their study, which was in consonance with the results obtained from catabolic gene probe analysis and DGGE analysis of the rhizosphere microbial community. Zucchi et al. (2003) used CO_2_ evolution from a crude oil-polluted soil to monitor oil degradation in 1 l jars equipped with a 50-ml beaker containing 10 ml 1 M KOH to trap the evolved CO_2_. The trapped CO_2_ in the KOH solution was quantified by back titration with 1 M HCl using a radiometer. Their results also correlated positively with the microbiological/molecular data obtained using internal transcribed spacer homoduplex heteroduplex polymorphisms (ITS-HHP) fingerprinting of total soil bacterial DNA and PCR-amplification of the catechol-2,3-dioxygenase (C23O) genes. Amouric et al. (2006) monitored the biodiversity of hexadecane-degrading consortium in a biofilter using CO_2_ evolution. They measured CO_2_ concentration contained in the outlet of the biofilter with thermal conductivity detector gas chromatograph. Their results confirmed that the isolated consortium belonging to *Actinomycetes* demonstrated high degradation of hexadecane as also recorded using DNA base composition and DNA–DNA hybridization studies. Hamamura et al. (2006) also used the production of ^14^CO_2_ from ^14^C-hexadecane to study the microbial population dynamics associated with crude oil biodegradation in diverse soils. Their findings showed that specific bacterial populations were responsible for the degradation of the hydrocarbons in the crude oil as recorded using radiorespirometry and DGGE analysis of PCR-amplified 16S rRNA gene.

## Biochemical methods

### Community level physiological profiling (CLPP)

Community level physiological profiles provide an indication of the metabolic diversity/fingerprint (substrate utilization pattern) present in an environment with respect to the number of defined substrates that can be oxidized by the autochthonous bacteria found there (Maila 2005). Basically, here the diversity unit is the ability of the whole microbial community to degrade specific substrates (range of sugars, carboxylic acids, amino acids and peptides) to provide an unparalleled wealth of discriminating biochemical characterizations. The community level carbon source utilization pattern obtained can be analyzed by applying the Biolog (Biolog, Inc., Hayward, CA, USA; http://www.biolog.com) tetrazolium-based redox dye technology. The method uses 96-well microplates/ecoplates containing 31 or 95 different carbon sources (Torsvik and Øvreås 2007) for Gram-negative and Gram-positive bacteria. The characteristic substrate utilization pattern or ‘metabolic fingerprint’ produced by the microbial community extracted from the soil or any other environmental sample can thus provide a measure of the community metabolic potential. Pure cultures can as well be identified using different Biolog automated systems coupled with a wide range of identification profiles/databases of different reference microorganisms. Such systems include the MicroLog1 (manual system) MicroStation ID (semi automated system) and OmniLog ID (fully automated) systems (http://www.biolog.com). The results from the CLPP analysis can be further used to describe the community’s functional diversity using diversity indices like the Shannon–Weaver index or statistical tools like the principal component analysis (PCA) (Torsvik and Øvreås 2007). Miller et al. (2002) used CLPP to study the diversity and function of soil bacteria exposed to mercury and tylosin. Their results showed that these contaminants reduced the bacterial diversity which correlated with that obtained with DGGE. In the same vein, Bundy et al. (2004) used CLPP to monitor the recovery of an oil-polluted soil after bioremediation and reported that oil contamination reduced microbial diversity but increased catabolic diversity by selecting for competitive, generalists bacterial populations able to degrade hydrocarbons. Maila et al. (2006) investigated the influence of geographic location and different hydrocarbon pollutants on soil microbial communities using CLPP. They found out that all the hydrocarbon-contaminated soils from different locations clustered together having demonstrated similar substrate utilization patterns. They concluded that hydrocarbons rather than geographic location were more important in determining the functional or species diversity within bacterial communities in oil-contaminated soils. Despite the usefulness of the CLPP method in microbial diversity studies, it has shortcomings due to its dependence on culturing and the fact that it gives functional rather than structural information about microbial species in polluted environments (Bundy et al. 2004; Maila 2005).

### Phospholipids fatty acids (PLFA) analysis

Total phospholipids fatty acids extracted from environmental samples have been used to study microbial community structures and metabolic states and to compare similarities and differences among soil microbial communities (Ogram et al. 2007). Phospholipids are important components of living cell membranes and constitute a significant proportion of organism biomass under natural conditions (Kozdroj and van Elsas 2001). Odd numbered and branched-chain fatty acids are associated with Gram-positive bacteria, while Gram-negative bacteria on the other hand contain higher proportions of even numbered monosaturated straight chain and cyclopropane fatty acids, eubacteria generally do not contain polyunsaturated fatty acids and plasmalogen phospholipids are enriched in anaerobic prokaryotic bacteria (Ogram et al. 2007). Microorganisms have the ability to change the lipid composition of their membranes in response to environmental conditions such as chemical stress, pollution (Frostegard et al. 1993) and temperature fluctuations (Bartlett 1999). PLFA rapidly degrade upon cell death thus making it a good indicator of living organisms (Drenovsky et al. 2004), and changes in PLFA patterns under environmental stress conditions are a useful biomarker tool to describe the community structure and physiological state of certain microbial taxa (Vestal and White 1989; Misko and Germida 2002). Changes in phospholipid profiles are generally related to the variation in the abundance of microbial groups and this can be interpreted by reference to a database of pure cultures and known biosynthetic pathways (Zelles 1999). The extracted fatty acids are quantitatively analyzed by gas chromatography equipped with mass spectrometry (Zelles and Bai 1993), while comparison of data with information on fatty acids database allows for the identification of extracted PLFAs (Widmer et al. 2001). Although direct extraction of PLFA from soil does not permit delineation down to species level, it is an efficient means by which gross changes in microbial community structure can be profiled (Nannipieri et al. 2003). Several researchers have taken a variety of approaches to the interpretation of community fatty acids profiles (Haack et al. 1994, 2004). Methods such as tabulation of known or presumed unique fatty acids or comparisons of profiles on the basis of within profile ratios of fatty acids have been used (Haack et al. 1994). Currently, PLFA analysis employs the use of multivariate statistics such as PCA to discriminate between composite profiles (Haack et al. 1994; Langworthy et al. 1998; Macnaughton et al. 1999; van Elsas et al. 2007).

Frostegard et al. (1993) examined changes in microbial population profiles in soils artificially polluted with cadmium, copper, nickel, lead or zinc using PLFA. They observed that certain fatty acid patterns characteristic of Gram-positive bacteria were reduced in both forest and arable soils spiked with metals and replaced by PLFA patterns indicative of a Gram-negative bacterial populations. In another independent investigation by Macnaughton et al. (1999) on microbial population changes during the bioremediation of an experimental oil spill, microbial community structures were monitored by PLFA. The results of PLFA analysis demonstrated a community shift in all plots (oil-polluted and unpolluted) from primarily eukaryotic biomass to Gram-negative bacterial biomass with time. PLFA profiles from the oil-polluted plots suggested increased Gram-negative biomass and adaptation to metabolic stress compared to unpolluted controls. Kamaludeen et al. (2003) investigated the ecotoxicity of long-term tannery waste contaminated soils by assessing the bacterial activity and community structure using PLFA. PLFA profiles of specific bacteria decreased significantly as the level of chromium contamination increased, indicating that the concentration of chromium in tannery waste contaminated soil had a significant effect on microbial community structure (Kamaludeen et al. 2003). In another study, a change in microbial community structure during bioremediation of explosives-contaminated soil in a molasses-fed bioslurry process was demonstrated using PLFA profiles (Fuller and Manning 2004). PLFA analysis showed that Gram-positive bacterial populations were more abundant after explosives compounds were reduced to non-inhibitory levels (Fuller and Manning 2004). PLFA profiles have also been employed to characterize the microbial community in PAH-contaminated freshwater sediments (Langworthy et al. 1998). Characterization of sulfate-reducing bacteria in groundwater at a uranium mill (Chang et al. 2001) and the study of microbial community structure at uranium-contaminated groundwater sources (Schryver et al. 2006) have been studied using PLFA. Comparison of PLFA and 16S rRNA in a phylogenetic study of 25 isolates of dissimilatory sulfate-reducing bacteria showed highly congruent clustering for 22 isolates (Kohring et al. 1994), thus establishing the usefulness of PLFA in the determination of bacterial relationships. Bundy et al. (2002)used PLFA to study the comparative effect of diesel contamination and simulated bioremediation on the microbial community in different soil types. Their findings showed that there was no tendency for the community structure of the three different soil types to converge as a result of contamination with the same hydrocarbon, rather they became more dissimilar. In another related investigation, Margesin et al. (2007) studied the influence of diesel oil concentration, biostimulation with inorganic/oleophilic fertilizers and incubation time on hydrocarbon removal. Microbial communities as assessed by PLFA patterns were primarily influenced by hydrocarbon content and fertilization. Among bacteria, PLFA indicative of the Gram-negative population were significantly increased (*P* ≤ 0.05) in soil samples containing high concentrations of diesel that received NPK fertilizer. However, PLFA analyses are not without limitations as fatty acid composition can be influenced by temperature and nutrition (Graham et al. 1995). Furthermore, individual fatty acids cannot be used to represent specific species (a single microorganism can have numerous fatty acids and the same fatty acids can occur in more than one species) (Kirk et al. 2004). PLFA as a microbial community profiling tool produces profiles of limited complexity, thus PLFA is often used in conjunction with other molecular profiling methods to assess microbial diversity in contaminated soil and water (Ringelberg et al. 2001).

## Molecular methods

### Metagenomic era

Metagenomic approaches have enabled us to understand the genomic potential of the entire microbial community in an ecosystem by cloning and analyzing microbial community DNA directly extracted from environmental samples. The development of improved DNA extraction methods, cloning strategies, screening techniques and high throughput sequencing methods has led to the emergence of various bioinformatic tools for the analysis and comparison of metagenomic data set with respect to taxonomic and metabolic diversity (Delmont et al. 2011; Morales and Holben 2011; Simon and Daniel 2011).

Estimating the microbial diversity of environmental samples is a major challenge during bioremediation. Ever since the discovery of bacterial pure culture techniques by Robert Koch, microbiological culture techniques have been significantly improved. However, a majority of bacterial species in any environment are still unculturable in the laboratory, due to the lack of knowledge of the real conditions under which these bacteria grow in their natural environment. Unfortunately, only a fraction of the microorganisms involved in the biodegradation of contaminants in soil can currently be cultured in the laboratory. It has been estimated that the microbial community in 1 g of soil may contain over 1,000 different bacterial species, but <1% of these may be culturable. It has also been observed that fast growing organisms or strains best adapted to particular culture conditions grow preferentially than those which are not, and therefore do not accurately represent the actual microbial community composition of contaminated environments. Hence, culture-dependent characterization of microorganisms at contaminated sites may limit the scope of microbial biodiversity (Zengler 2008). The application of molecular techniques to study microbial populations at contaminated sites without the need for culturing has led to the discovery of unique and previously unrecognized microorganisms as well as complex microbial diversity in contaminated soil and water, which shows an exciting opportunity for bioremediation strategies. Nucleic acid extraction from contaminated sites and their subsequent amplification by PCR have proved extremely useful in assessing the changes in microbial community structure by several microbial community profiling techniques. Microbial diversity is considered as a function of the number of different classes (richness) and the relative distribution of individual elements among these classes (evenness). The diversity assessment is generally based on (1) PCR-amplification of 16S genes from metagenomic DNA; (2) making 16S gene libraries; (3) sequencing of randomly selected clones; and (4) phylogenetic analysis (Rajendhran and Gunasekaran 2011). The richness and evenness of a community are qualitatively estimated based on the number of unique clones and their relative frequencies. However, the validity of metagenomics-based microbial diversity analysis depends on obtaining representative nucleic acids from entire microbial community. The quality and quantity of the metagenomic DNA influences the microbial community structure. Incomplete cell lysis, DNA sorption to inert matrices, coextraction of enzymatic inhibitors and degradation of DNA at various steps of extraction procedures may influence the microbial diversity pattern. In addition, biases may be introduced during PCR-amplification and cloning steps (Malik et al. 2008). A summary of the molecular methods used in monitoring microbial community composition and dynamics during bioremediation is presented in Table 2.

### Analysis of taxonomic and functional diversity in microbial populations

Microbial diversity and dynamics in environments such as soil undergoing bioremediation have been assessed by the analysis of conserved marker genes such as 16S rRNA genes. In addition, large databases of reference sequences, such as Greengenes, SILVA or Ribosomal Database Project II (RDP II) (Simon and Daniel 2011), provide an important and useful resource for rRNA gene-based classification of microorganisms. In addition, other conserved genes such as *recA* or *radA* and genes encoding heat shock protein 70, elongation factor Tu or elongation factor G and hydrocarbon degradative enzymes (Peng et al. 2008; Flocco et al. 2009; Morales and Holben 2011) have been employed as molecular markers for phylogenetic analyses. The use of next-generation sequencing technologies, such as pyrosequencing of 16S rRNA gene amplicons, has provided unprecedented sampling depth compared to traditional approaches, such as denaturing gradient gel electrophoresis (DGGE), terminal restriction fragment length polymorphism (T-RFLP) analysis or Sanger sequencing of 16S rRNA gene clone libraries (Rajendhran and Gunasekaran 2011). However, the intrinsic error rate of pyrosequencing may result in the overestimation of rare phylotypes. Each pyrosequencing read is treated as a unique identifier of a community member, and correction by assembly and sequencing depth, which is typically applied during genome projects, is not feasible. A crucial step in the taxonomic analysis of large metagenomic data sets is called binning. Within this step, the sequences derived from a mixture of different organisms are assigned to phylogenetic groups according to their taxonomic origins. Depending on the quality of the metagenomic data set and the read length of the DNA fragments, the phylogenetic resolution can range from the kingdom to the genus level. Currently, two broad categories of binning methods can be distinguished: similarity-based and composition-based approaches. The similarity-based approaches classify DNA fragments based on sequence homology, which is determined by searching reference databases using tools like the Basic Local Alignment Search Tool (BLAST). Examples of bioinformatic tools employing similarity-based binning are the Metagenome Analyzer (MEGAN), CARMA, or the sequence ortholog-based approach for binning and improved taxonomic estimation of metagenomic sequences (Sort- ITEMS). CARMA assigns environmental sequences to taxonomic categories based on similarities to protein families and domains included in the protein family database (Pfam), whereas MEGAN and Sort-ITEMS classify sequences by performing comparisons against the NCBI nonredundant and NCBI nucleotide databases (Simon and Daniel 2011). These approaches are currently employed in microbial monitoring during hydrocarbon degradation and have been shown to yield resounding results. Surridge (2007) used PCR-DGGE, sequencing of 16S, *xylE* and *ndoB* gene fragments (encoding dioxygenases) to characterize microbial communities in PAHs and polychlorinated biphenyl (PCB) contaminated soil. According to GenBank identification using BLAST, the bacteria *Sphingomonas adhaesiva*, *Sphingomonas terrae*, *Sphingopyxis witflariensis*, *Sphingomonas* sp., *Methylocystis* sp., *Pseudomonas* sp., *Pseudomonas marginalis*, *Acidocella* sp. and *Acidiphilium facilis* were among the dominant sequences in the PAH and PCB-polluted soils. Phylogenetic analysis with dendograms constructed using CLUST software also revealed diversity of aromatic hydrocarbon degradative genes that clustered the genera *Burkholderia*, *Sphingomonas*, *Pseudomonas*, *Bacillus*, *Methylobacterium*, *Klebsiella* and *Rhodococcus* as well as *Vibrio* together, thus describing the production of catechol 2,3 dioxygenase and naphthalene dioxygenase. By supplementing phylogenetic-based community dynamics tools with analysis of microbial community composition and diversity using specific catabolic gene markers, clearer information can be obtained on the behavior of the organisms that are specifically responsible for degrading hydrocarbons, free from variation in members of the community that do not directly participate in hydrocarbon degradation.

## Conclusion

The clear-cut means for evaluating bioremediation of hydrocarbon-contaminated soils is a direct measurement of hydrocarbon dissipation or the residuum. However, the success of a bioremediation project can often be estimated by measuring the biodegradability of the contaminants. Because biodegradation is a microbial-driven process, it can be indirectly assessed by measuring the microbial numbers, biomass and/or activity. Methods that are used include enumeration of hydrocarbon degraders using any hydrocarbon as a selective pressure, measurement of community metabolic activities by respirometry or Biolog system, degradative enzyme-based assays, metagenomic/nucleic acid-based techniques and phospholipid fatty acid analysis. Proper application of these strategies and techniques assists the investigators to monitor population dynamics during the course of bioremediation.
